# Registered Influenza Viral Vector Based *Brucella abortus* Vaccine for Cattle in Kazakhstan: Age-Wise Safety and Efficacy Studies

**DOI:** 10.3389/fcimb.2021.669196

**Published:** 2021-07-01

**Authors:** Sholpan Ryskeldinova, Nadezhda Zinina, Zhailaubay Kydyrbayev, Bolat Yespembetov, Yerken Kozhamkulov, Dulat Inkarbekov, Nurika Assanzhanova, Aigerim Mailybayeva, Dina Bugybayeva, Makhpal Sarmykova, Berik Khairullin, Kairat Tabynov, Aitbay Bulashev, Batyrbek Aitzhanov, Khairulla Abeuov, Abylay Sansyzbay, Tlektes Yespolov, Gourapura J. Renukaradhya, Steven Olsen, Angel Oñate, Kaissar Tabynov

**Affiliations:** ^1^ Infectious Disease Prevention Laboratory, Research Institute for Biological Safety Problems, Gvardeiskiy, Kazakhstan; ^2^ Microbiology Laboratory, Research Institute for Biological Safety Problems, Gvardeiskiy, Kazakhstan; ^3^ International Center for Vaccinology, Kazakh National Agrarian University (KazNAU), Almaty, Kazakhstan; ^4^ Infectious Disease Monitoring Laboratory, Research Institute for Biological Safety Problems, Gvardeiskiy, Kazakhstan; ^5^ Preclinical Research Laboratory With Vivarium, M. Aikimbayev National Research Center for Especially Dangerous Infections, Almaty, Kazakhstan; ^6^ Department of Microbiology and Biotechnology, S. Seifullin Kazakh Agrotechnical University, Nur-Sultan, Kazakhstan; ^7^ Department of Clinical Veterinary Medicine, Kazakh National Agrarian University (KazNAU), Almaty, Kazakhstan; ^8^ Department of Biological Safety, Kazakh National Agrarian University (KazNAU), Almaty, Kazakhstan; ^9^ Food Animal Health Research Program, Ohio Agricultural Research and Development Center, Department of Veterinary Preventive Medicine, The Ohio State University (OSU), Wooster, OH, United States; ^10^ Independent Researcher, McCallsburg, IA, United States; ^11^ Laboratory of Molecular Immunology, Department of Microbiology, Faculty of Biological Sciences, University of Concepcion, Concepcion, Chile

**Keywords:** bovine brucellosis, influenza viral vector, vaccine, registration trials, protective efficacy, calves, heifers, cows

## Abstract

A novel influenza viral vector based *Brucella abortus* vaccine (Flu-BA) was introduced for use in cattle in Kazakhstan in 2019. In this study, the safety and efficacy of the vaccine was evaluated in male and female cattle at different ages, and during pregnancy as a part of its registration process. Our data demonstrated that the Flu-BA vaccine was safe after prime or booster vaccination in calves (5–7 months old male and female), heifers (15–17 months old) and cows (6–7 years old) and was not abortogenic in pregnant animals. A mild, localized granuloma was observed at the Flu-BA injection site. Vaccinated animals did not show signs of influenza infection or reduced milk production in dairy cows, and the influenza viral vector (IVV) was not recovered from nasal swabs or milk. Vaccinated animals in all age groups demonstrated increased IgG antibody responses against *Brucella* Omp16 and L7/L12 proteins with calves demonstrating the greatest increase in humoral responses. Following experimental challenge with *B. abortus* 544, vaccinates demonstrated greater protection and no signs of clinical disease, including abortion, were observed. The vaccine effectiveness against *B. abortus 544* infection was 75, 60 and 60%, respectively, in calves, heifers and adult cows. *Brucella* were not isolated from calves of vaccinated cattle that were experimentally challenged during pregnancy. Our data suggests that the Flu-BA vaccine is safe and efficacious in cattle, including pregnant animals; and can therefore be administered to cattle of any age.

## Introduction

*Brucella melitensis*, *Brucella abortus*, *B. suis* and *B. canis* are all considered to be zoonotic (O’Callaghan and Whatmore, 2011). Brucellosis is one of the most common zoonotic diseases of humans, with more than 500,000 cases reported annually. Depending upon the system of controls and the socioeconomic conditions, different countries have reported from 0.09 to 1,603 cases per million inhabitants (Pappas et al., 2006).

*B. abortus* is the primary cause of brucellosis of cattle. Because of its impact on human health, in Kazakhstan, regulatory actions for cattle herds infected with brucellosis include quarantine (Yespembetov et al., 2019). In cattle, brucellosis can be manifested by orchitis in males, but clinical signs are primarily in females and include nonviable calves, abortions, retained placentas, and infertility (O’Callaghan and Whatmore, 2011). Vaccination is an effective tool for controlling brucellosis in livestock (Garin-Bastuji et al., 1998), and is also effective in protecting human health in endemic areas (Zinsstag et al., 2007). Currently, brucellosis vaccines for cattle are live attenuated *B. abortus* strains (19, 82 and RB51). Although these vaccines have high efficacy for cattle (protection against abortion >70%, complete protection against infection >50%) (Confer et al., 1985; Stevens et al., 1995; Cheville et al., 1996; Cardena et al., 2009; Ivanov et al., 2011), they have a number of serious disadvantages including causing abortions in pregnant animals, virulence in humans, and, with the exception of strain RB51, cause high titers on brucellosis serologic tests that cannot be differentiated from responses of infected animals (Spink et al., 1962; Beckett and MacDiarmid, 1985; Smith and Ficht, 1990). Additionally, the strain RB51 is resistant to rifampicin, an antibiotic commonly used to treat brucellosis in humans (Schurig et al., 1991). These characteristics of commercial vaccines have limited their wide use in cattle in some countries. Development of an improved brucellosis vaccine for cattle with high efficacy, improved safety characteristics, and the ability to be serologically differentiated from infected animals (DIVA) would be an important advancement.

Previously, attempts to develop safe and effective *B. abortus* vaccines have utilized attenuated mutants (Vemulapalli et al., 2000a; Vemulapalli et al., 2000b; Vemulapalli et al., 2004; Olsen et al., 2009), subunit (recombinant proteins) vaccines (Tabatabai and Pugh, 1994; Oliveira et al., 1994a; Oliveira et al., 1994b; Oliveira and Splitter, 1996; Oliveira et al., 1996; Kurar & Splitter, 1997; Al-Mariri et al., 2001; Cassataro et al., 2005a; Mallick et al., 2007; Pasquevich et al., 2009), DNA vaccines (Leclercq et al., 2003; Oñate et al., 2003; Cassataro et al., 2005b; Luo et al., 2006), RNA vaccine (Oñate et al., 2005) and vector-based vaccines (He et al., 2002; Cabrera et al., 2009; Zhao et al., 2009). The above mentioned vaccine candidates induced antigen-specific Th1 immune responses, and demonstrated protection against brucellosis challenge that was comparable to commercial attenuated vaccine strains (*B. abortus* S19 or RB51) (Tabatabai and Pugh, 1994; Oliveira et al., 1994a; Oliveira et al., 1994b; Oliveira and Splitter, 1996; Oliveira et al., 1996; Kurar and Splitter, 1997; Vemulapalli et al., 2000a; Vemulapalli et al., 2000b; Al-Mariri et al., 2001; He et al., 2002; Leclercq et al., 2003; Oñate et al., 2003; Vemulapalli et al., 2004; Cassataro et al., 2005a; Cassataro et al., 2005b; Oñate et al., 2005; Luo et al., 2006; Mallick et al., 2007; Cabrera et al., 2009; Olsen et al., 2009; Pasquevich et al., 2009; Zhao et al., 2009). However, the vaccine candidates were usually not tested in large animal host species, such as cattle. With the exception of the double mutant strain *B. abortus* htrA cycL vaccine; and DNA and RNA vaccine expressing Cu-Zn superoxide dismutase gene which tested for immunogenicity in cattle, but not efficacy (Edmonds et al., 2000; Sáez et al., 2008). A recombinant *B. abortus* RB51 vaccine (overexpressing superoxide dismutase and glycosyltransferase genes) was immunogenic in bison but not as efficacious as the parental RB51 vaccine against challenge with the *B. abortus* strain 2308 (Olsen et al., 2009). Consequently, most *B. abortus* vaccine candidates have limited or no data available on their safety and efficacy in cattle, and therefore, are not available for field use.

With the goal of improving prevention of bovine brucellosis, our group developed a novel vaccine based on an influenza viral vector (IVV) platform, which was registered and permitted for field use in Kazakhstan in 2019. We used influenza A viruses of various subtypes as carriers for delivery of immunodominant proteins of *Brucella*. Influenza viruses were chosen as a vector because of their ability to infect cattle without causing clinical signs (Campbell et al., 1977; Brown et al., 1998; Gunning et al., 1999; Graham et al., 2002). The influenza A virus contains a segmented genome consisting of eight negative-strand RNA fragments. Of these, the smallest fragment (NS), encoding two proteins: viral nonstructural protein (NS1) and nuclear export protein (Nep), is a convenient target for genetic manipulation since NS1 tolerates foreign sequences exceeding its own length (Kittel et al., 2004). On the other hand, *Brucella* constructs encoding 124 amino acids from the N-terminal of the *B. abortus* immunodominant L7/L12 and Omp16 proteins (Oliveira et al., 1994a; Tibor et al., 1999) were inserted into the NS1 gene of the A/Puerto Rico/8/34 (H1N1) influenza strain. The vectors were constructed using the surface hemagglutinin (HA) and neuraminidase (NA) glycoproteins from the A/chicken/Astana/6/05 (H5N1) virus (with removed HA cleavage site) and seasonal А/New Caledonia/20/99 (H1N1) virus.

In previous studies, we demonstrated that the IVV based *B. abortus* vaccine (Flu-BA) was safe and effective in cattle (including pregnant animals) (Tabynov et al., 2014a; Tabynov et al., 2016a), and induced humoral and cell-mediated immune responses (Tabynov et al., 2014d; Tabynov et al., 2014e). Conjunctival or subcutaneous vaccination of pregnant heifers induced protection against infection after *B. abortus* 544 challenge in 70–80% and prevented abortions in 80-90% of animals that was comparable to protection after vaccination with *B. abortus* S19 (Tabynov et al., 2014e). Simultaneous conjunctival and subcutaneous vaccination with Flu-BA vaccine induced 100% protection against abortion and 88.8 to 100% protection against infection after *B. abortus* 544 challenge which was greater than protection by a commercial *B. abortus* strain S19 vaccine (Tabynov et al., 2016b). As the Flu-BA vaccine does not form *Brucella* lipopolysaccharide, no antibodies are induced in vaccinated cattle thereby meeting the DIVA criteria (Tabynov et al., 2016a). Data suggests that the Flu-BA vaccine in cattle provides protective immune responses against *B. abortus* infection for at least 12 months after booster vaccination (Tabynov et al., 2016c) and provides cross protection against *B. melitensis* (Tabynov et al., 2015). *Brucella melitensis* can be transmitted from small ruminants to cattle in multi-species farms and has been reported in a number of countries (Verger et al., 1989; Alvarez et al., 2011).

Although the Flu-BA vaccine has been evaluated in cattle from 17 months to 2 years of age, including pregnant cattle, however, the safety and efficacy of the vaccine not been evaluated in a broader age of cattle. Previous studies have not evaluated Flu-BA vaccinated cattle for signs of influenza infection. The current study was designed to compare responses to vaccination in a broader age range of cattle.

This study was carried out in accordance with the program of registration commission testing of safety and protectiveness of the vector based vaccine against bovine brucellosis, approved by the National Veterinary Reference Centre on June 20, 2018, as well as according to the order of the Chairman of the Veterinary Control and Supervision Committee of the Ministry of Agriculture of the Republic of Kazakhstan (#76 dated May 24, 2018). Studies were conducted in full compliance with national and international animal welfare laws/guidelines. The protocol was approved by the Committee on the Ethics of Animal Experiments of the Research Institute for Biological Safety Problems of the Science Committee of the Ministry of Education and Science of the Republic of Kazakhstan.

## Materials and Methods

### Bacterial Strains

The virulent strain *B. abortus 544* (obtained from Research Institute for Biological Safety Problem) were used in this study. The bacterial cells were cultured under aerobic conditions in *Brucella* Base agar (Sigma, St. Louis, MO, USA) at 37°C. All experiments with live *Brucella* were performed in biosafety level 3 facilities.

### Influenza Viral Vectors

Influenza viral vectors (IVV) were generated in HSC Development GmbH (Tulln, Austria) by a standard reverse genetics method using eight bidirectional plasmids pHW2000 as described previously (Tabynov et al., 2014b). Schematic representation of the recombinant influenza viral vector construction was described previously (Bugybayeva et al., 2021). Briefly, Vero cells were co-transfected by LonzaNucleofector™ (Cologne, Germany) technique with 0.5 μg/μl of plasmids encoding the PB1, PB2, PA, NP, M gens and NS (chimeric) gene of А/Puerto Rico/8/34 (H1N1) virus; and the HA and NA genes of A/chicken/Astana/6/05 (H5N1) or А/New Caledonia/20/99 (H1N1) strains. The HA protein sequence of the H5 virus was attenuated by means of exchanging its polybasic cleavage site to one containing a trypsin-dependent sequence. The NS genes were modified to express NS1 fusion proteins containing a sequence of 124 N-terminal amino acids from the NS1 protein coupled with a sequences of *B. abortus* derived proteins: L7/L12 (GenBank: AAA19863.1) or Omp16 (GenBank: AAA59360.1), ended with double stop codon. *Brucella* sequences were obtained synthetically. The supernatants of transfected cells were used for inoculation into 10-day-old chicken embryos (CE; Lohmann Tierzucht GmbH, Cuxhaven, Germany]) which was incubated at 34°C for 48 h. Vaccine batches were produced in CE after three egg passages of viral constructs. A total of four IVV of the subtypes H5N1 or H1N1 expressing the *Brucella* L7/L12 or Omp16 proteins from the ORF of the NS1 gene were generated: H5N1 (Flu-NS1-124-L7/L12-H5N1, Flu-NS1-124-Omp16-H5N1) and H1N1 (Flu-NS1-124-L7/L12-H1N1 and Flu-NS1-124-Omp16-H1N1).

### Vaccine Formulation

The vaccine formulation was prepared with IVV Flu-NS1-124-L7/L12-H5N1, Flu-NS1-124-Omp16-H5N1, Flu-NS1-124-L7/L12-H1N1 and Flu-NS1-124-Omp16-H1N1; the IVV were reproduced in 10-day-old chicken embryos (CE) at 34°C for 48 h. The titer of the IVV was determined in CE, as previously described (Tabynov et al., 2014c; Tabynov et al., 2015). Briefly, the obtained allantoic suspensions of viral constructs with the same antigenic structure (H5N1 or H1N1) were combined in a single pool in a 1:1 ratio to obtain the bivalent vaccine formulation. Then the mixtures of IVV (L7/L12 + Omp16) were mixed in a 1:1 ratio with sterile stabilizing medium containing 12% peptone from casein (Sigma-Aldrich) and 6% sucrose (Sigma-Aldrich), mixed, aliquoted into 1 ml ampoules, lyophilized and stored at 4 °C. Flu-BA vaccine prototype consisting of IVV subtype H5N1 was marked as “Vaccine 1” (batch 1, 29 June 2018, 20 doses/ampoule, produced by RIBSP) and used for prime vaccination. Vaccine samples consisting of IVV subtype H1N1 were marked as “Vaccine 2” (batch 1, 29 June 2018, 20 doses/ampoule, produced by RIBSP) and used for booster vaccination. When necessary, the lyophilizate was resuspended in 20% Montanide™ Gel-01 (Seppic, France) adjuvant in PBS, sterilized by autoclaving between 120 and 130°C, and aliquoted and sealed in 50 ml of bottles.

For immunization, three ampoules from each vaccine batch were resuspended to their original volume with a diluent (20% adjuvant Montanide Gel 01 in PBS) and combined. From the combined material, 2 ml of vaccine was dissolved in 18 ml of preparation solvent (20 doses, each dose of 1 ml). Animals were vaccinated with 2 ml of the diluted vaccine.

### Animals

In the research, cattle of the Kazakh white-headed breed obtained from a breeding farm in Kazakhstan free from brucellosis for the past 10 years were used. Animals were brought to the experimental animals wing of the Research Institute for Biological Safety Problem, and were tested twice at intervals of 14–28 days by serological methods to confirm the absence of brucellosis.

### Vaccination and Study Design

Kazakh white-headed breed cattle were divided into three groups (n = 10/group) by sex, age, and pregnancy status. Group I included equal numbers of male and female calves at 5 to 7 months of age. Group II were heifers at 15–17 months of age and included pregnant animals at 1.5 to 3 months of gestation (n = 4) at the time of inoculation. Pregnancy was confirmed by using ultrasound and rectal palpation methods. Group III consisted of 6 to 7-year-old cows and included pregnant animals at 1.5- to 4 months gestation (n = 6) at the time of inoculation. Animals in each of these groups were randomly divided into two subgroups (**Table 1**), vaccinated and controls (n = 5/group). Pregnant animals in groups II and III were equally divided between control and vaccine treatments. Vaccinated calves, heifers and adult cows (n = 5 per age group) were subcutaneously immunized twice in the cervical region at a 28-day interval with vaccines generated from the IVV subtypes H5N1 (prime vaccination; 6.2–6.5 log_10_ EID_50_/animal) and H1N1 (booster vaccination; 6.1–6.3 log_10_ EID_50_/animal). Animals in the control group (n = 5 per age group) were subcutaneously injected with 1.0 ml of 20% Montanide Gel01 adjuvant in PBS. Vaccine safety was assessed by monitoring local adverse reactions, and signs of influenza infection (nasal effluents, coughs, reduced milk production, persistence of IVV in polymerase chain reaction) twice per day for 28 days after each vaccination (56 days in total). On day 28 after prime and booster vaccination, blood samples were collected from all animals to evaluate antibody responses to *Brucella* LPS, influenza antigens (type A and subtypes H5 and H1), and *Brucella* Omp16 and L7/L12 proteins.

**Table 1:** Randomized cattle groups.

Group	Subgroup	No.	Animal sex	Inventory number	Age	Pregnancy, months
**I Calves**	Vaccinated	1	Female	60217909	6 months	–
		2	Male	60112522	7 months	–
		3	Male	60217910	6 months	–
		4	Female	60112532	7 months	–
		5	Female	60217907	7 months	–
	Control	6	Female	60112520	7 months	–
		7	Male	60112533	7 months	–
		8	Male	60112524	6 months	–
		9	Male	60486298	7 months	–
		10	Female	60456657	5 months	–
**II Heifers**	Vaccinated	11	Female	99625616	16 months	1.5
		12	Female	59631156	15 months	n/p
		13	Female	99625619	16 months	n/p
		14	Female	91190062	16 months	n/p
		15	Female	58033684	17 months	3.0
	Control	16	Female	00006547	16 months	1.5
		17	Female	91221247	16 months	n/p
		18	Female	59619127	15 months	n/p
		19	Female	60015348	17 months	n/p
		20	Female	60017619	16 months	2.0
**III Cows**	Vaccinated	21	Female	59074345	6 years	1.5
		22	Female	58034698	7 years	n/p
		23	Female	58034684	6 years	2.5
		24	Female	00184723	7 years	n/p
		25	Female	58034689	7 years	2.5
	Control	26	Female	59074333	6 years	2.0
		27	Female	58034825	7 years	n/p
		28	Female	58034696	6 years	4.0
		29	Female	59073052	6 years	1.5
		30	Female	59233164	7 years	n/p

n/p, non-pregnant.

**Figure 1:**
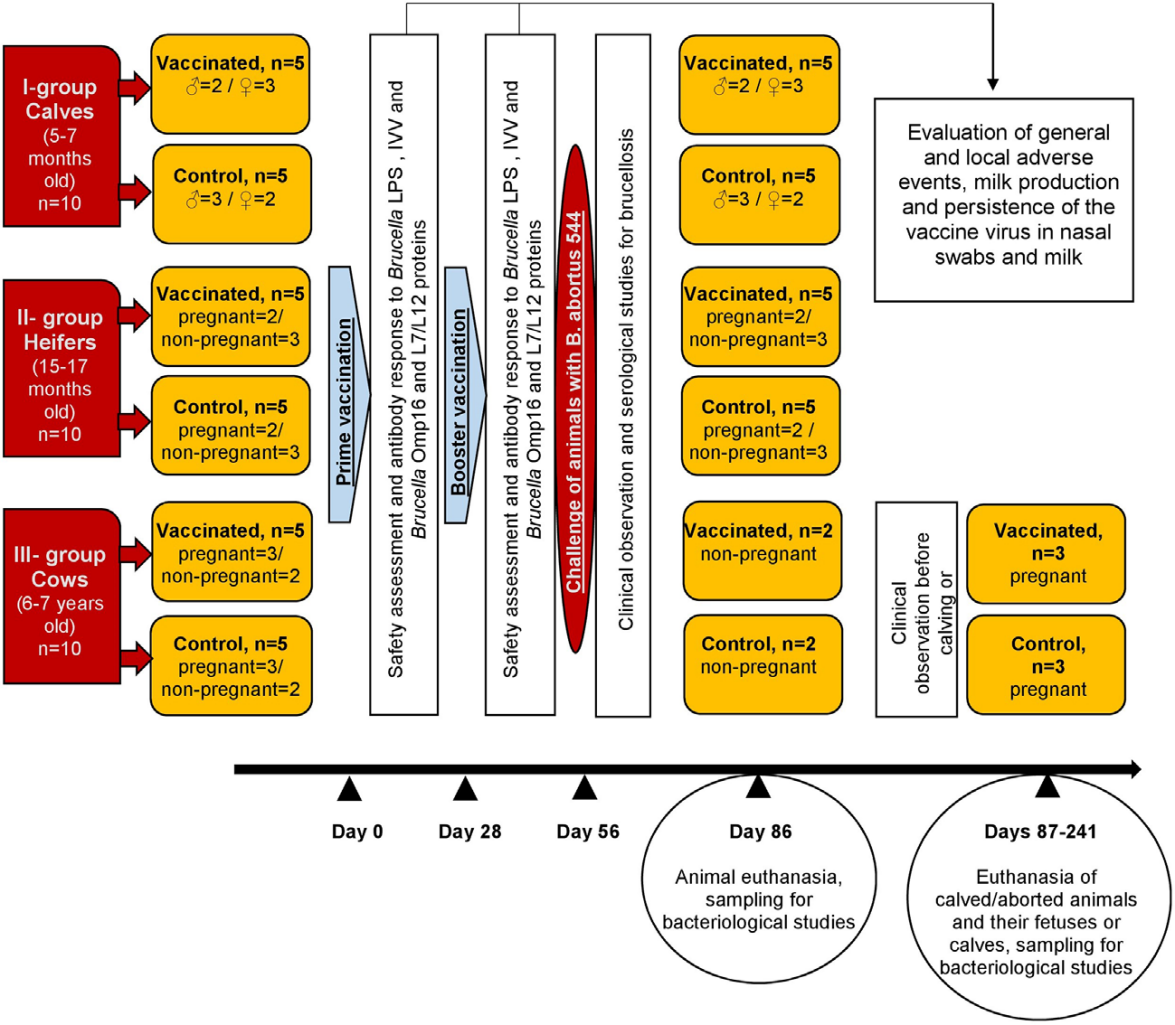
Experimental design and vaccination.

Vaccine efficacy was evaluated by experimental challenge of all animals with *B. abortus* 544 at 28 days of booster vaccination. All cattle were housed in a specialized biosafety level 3 agricultural facility for 21 days after booster vaccination. After 7 days of acclimation (28 days after booster vaccination), all animals were subcutaneously inoculated with approximately 5 × 10^8^ CFU/animal of *B. abortus* 544 in the middle third of the neck. Vaccinated and control animals were housed separately after challenge. Animals were provided with constant access to water, as well as a standard balanced diet. Blood was obtained from the jugular vein at 7, 14, 21, and 28 days after challenge for evaluation on Rose Bengal, Standard tube, and ELISA tests. Rectal temperature was assessed daily for 21 days after challenge. Animals in Groups I and II, and non-pregnant cattle (n = 4) in Group III were euthanized with sodium pentobarbital and necropsied at 30 days after challenge. Pregnant animals in Group III were euthanized with sodium pentobarbital and necropsied at calving or abortion after challenge. In animals necropsied at 30 days after experimental challenge, samples of lymph nodes (submandibular, retropharyngeal, right and left subscapular, right and left inguinal, mediastinal, bronchial, portal, paraortic, pelvic, mesenteric, udder), organs (liver, kidney, spleen), testicles (in males), and bone marrow were obtained for bacteriological studies. In animals challenged during pregnancy, placentome, placental fluid, fetal stomach contents. The design of the study was shown in **Figure 1**.

### Vaccine Safety Study

#### Clinical Observation

Animals were monitored twice daily for 56 days (28 days after prime and booster vaccinations). Clinical observations assessed include the general (overall condition, behavior, appetite, abortions in pregnant animals, temperature reaction within 0–7 and 21–27 days after each vaccination), local reactions (swelling/infiltrates at the administration site and its diameter, duration of swelling desorption) and adverse events detected in animals after each vaccination. In addition, the presence of influenza-like symptoms (nasal effluents, coughs, reduced milk productivity in dairy cows) in vaccinated animals was evaluated.

Skin thickness at the vaccination injection site was monitored using a caliper and expressed in mm. Diameter of inflammation both laterally and vertically was measured and an average thickness was calculated based on the average of obtained values. Skin reaction were monitored until no inflammation could be detected.

Milk production was monitored for 27 days after prime and booster vaccination in both vaccinated and non-vaccinated cattle (n = 3/group) after twice daily machine milking (Sezer Sağım Teknolojileri, Turkey). Total volume of milk per day was compared as a percentage of production prior to vaccination.

#### Assessing the Vaccine Viruses in Vaccinated Animals

Nasal swabs were collected from all animals in vaccinated and control groups at days 1 to 5 and at 21 days after prime and booster vaccination. In lactating animals, milk samples were also obtained. Nasal swabs were placed into tubes containing 1 ml of viral transport medium (phosphate-buffered saline containing 40% glycerol and 2% antibiotic solution). All samples were subsequently examined in a reverse transcriptional polymerase chain reaction (RT-PCR) to the influenza A virus as described (Chervyakova et al., 2011) using specific primers (sense 5’-ATGGTGCAGGCAATGAGG-3’ and antisense 5’-CAAGATCCCAATGAT-3’), and were used for viral isolation (with two consecutive passages) in 10-day CE (Alel Agro, Kazakhstan) as described (Tabynov et al., 2014c) with a few modifications. In brief, for virus isolation, the nasal swab samples were clarified by centrifugation at 3.000×*g* for 10 min and by filtration through 0.2 µm filter. The filtered material and milk samples were injected to CE allantoic cavity in dose of 0.2 ml and incubated at 34°C for 48 h and subjected to ovoscopy daily. The virus presence in CE after cooling (at 4°C for 16 h) was determined by hemagglutination (HA) assay (Tabynov et al., 2014c). The specificity of HA was determined using the commercial Directigen Flu A rapid assay (Becton Dickinson, Franklin Lakes, NJ, USA). Allantoic fluid samples were used in the same way collected after second passage in CE.

#### Determination of Immune Response to Influenza Viral Vectors

Blood samples were collected from all calves, heifers and cows of the experimental and control groups at 28 days after prime and booster vaccination. IgG antibodies specific to influenza type A virus were measured using a commercial ELISA kit (AniGen AIV Ab ELISA, Kyonggi-do, Korea) in accordance with manufacturer’s instructions. Humoral responses were also measured using a hemagglutination inhibition (HAI) assay as described previously (Tabynov et al., 2014c). ELISA results were considered positive for optical density (OD) inhibition >50% [(1 − (OD sample − OD negative control)) × 100]. HAI assay was performed by using 1% chicken red blood cell suspension. To remove non-specific inhibitors, blood samples were treated with the receptor-destroying enzyme from Vibrio cholera (Denka Seiken Co. Ltd., Japan). The native influenza viral vectors subtypes H5N1 or H1N1 were used as the antigen at the working dose of four hemagglutination units.

### Differentiation of Infected From Vaccinated Animals (Post-Vaccination Period)

Serum obtained at 28 days after prime or booster vaccination were tested for *Brucella* antibodies using the Rose Bengal test (RBT; Cenoqenics Corporation, USA), serum agglutination test (SAT; Microgen, Moscow, Russia), enzyme-linked immunosorbent assays (ELISA; Brucella-Ab C-ELISA, Svanova Biotech AB, Sweden), and milk ring test (Vivat, Ukraine) in accordance with manufacturer’s instructions. Responses were considered positive if: pronounced agglutination was present on the RBT (on two to four crosses); agglutination was detected in the SAT when serum was diluted ≥1:50; with inhibition of OD >30% on the ELISA [(OD sample − OD negative control/OD positive control − OD negative control] × 100); and detection of agglutination on the milk ring test (one to three crosses].

### Determination of *Brucella* Antigen-Specific Immune Response

Serum samples collected from all animals at 0 and 28 days after prime and booster vaccination were tested by ELISA for the presence of antibodies to *Brucella* Omp16 and L7/L12 proteins as previously described (Tabynov et al., 2016c). Briefly, 96-well microtiter plates (Nunc, Roskilde, Denmark) were coated overnight with pretitrated mixture, as well as individual *Brucella* L7/L12, or Omp16 proteins (each at 2 μg/ml) in PBS, blocked for 1 h using PBS containing 1% ovalbumin (PBS-OVA; 200 μl/well), and washed with PBS containing 0.05% Tween-20 (PBS/Tw). A serum sample of 100 μl/well was added to plates and incubated for 1 h at room temperature. Horseradish peroxidase-conjugated mouse anti-bovine IgG monoclonal antibody (mAb; clone IL-AR; Serotec, Raleigh, NC, USA) was used for detection. After a 90 min incubation at 37°C and washing, specific reactivity was determined by the addition of an enzyme substrate, ABTS [2,2-azinobis (3-ethylbenzthiazolinesulfonic acid)] diammonium (Moss, Inc., Pasadena, CA, USA) at 100 µl/well. The absorbance values were measured at 450 nm and expressed as optical density (OD).

### Determination of Vaccine Protectiveness

Vaccine efficacy was evaluated by experimental challenge of all animals with *B. abortus* 544 at 28 days of booster vaccination. All cattle were moved into a specialized biosafety level 3 agricultural facility at 21 days after booster vaccination. After 7 days of acclimation (28 days after booster vaccination), all animals were subcutaneously inoculated with approximately 5 × 10^8^ CFU/animal of *B. abortus* 544 in the cervical region. Vaccinated and control animals were housed separately after challenge. Blood was obtained from the jugular vein at 7, 14, 21, and 28 days after challenge for evaluation on Rose Bengal, Standard tube, and ELISA tests. Rectal temperature was assessed daily for 21 days after challenge. Animals in Groups I and II, and non-pregnant cattle (n = 4) in Group III were euthanized with sodium pentobarbital and necropsied at 30 days after challenge. Pregnant animals in Group III were euthanized with sodium pentobarbital and necropsied within 12 h of calving or abortion.

In animals necropsied at 30 days after experimental challenge, samples of lymph nodes (submandibular, retropharyngeal, right and left subscapular, right and left inguinal, mediastinal, bronchial, portal, paraortic, pelvic, mesenteric, udder), organs (liver, kidney, spleen), testicles (in males), and bone marrow were obtained for bacteriological studies. Additional samples from pregnant animals in Groups II and III included placentome, placental fluid, fetal stomach contents. Samples obtained from aborted fetuses and calves included: submandibular, retropharyngeal, right subscapular, left subscapular, right inguinal, mediastinal, portal, paraortic, pelvic, mesenteric lymph nodes, liver, kidney, spleen, and bone marrow.

Tissue samples were weighed, homogenized in 0.1% Triton–PBS, and 100 μl aliquots of 10-fold serial dilutions were plated in triplicate onto *Brucella* Base agar (Sigma, St. Louis, MO, USA) plates supplemented with 10% horse serum (Sigma, St. Louis, MO, USA) and *Brucella* Selective Supplement (Biolab Diagnostics Laboratory Inc., Budapest, Hungary) according to the manufacturer’s instructions. Plates were incubated at 37°C for 2 weeks, and periodically evaluated. The growth of *Brucella* cultures in plates was counted twice, the first time after 7 days and the second time after 14 days. After the first counting plates were moistened with sterile physiological solution to prevent the plates from drying. The animal was considered infected if *Brucella* was recovered from any sample. Bacteriological data were evaluated by: (А) Percentage of animals without recovery of *Brucella* at necropsy; (B) Infection index (number of samples from which *Brucella* was isolated); (C) Intensity/severity of *Brucella* colonization from samples (expressed as Log10 CFU/g of tissue). Vaccine effectiveness was determined as described previously (Cohen et al., 2012).

### Statistical Analysis

Milk productivity, dynamics of infiltrate resorption, antibody responses to *Brucella* LPS, IVV (IgG and HAI), *Brucella* Omp16 or L7/L12 proteins (IgG), the index of infection and colonization of *Brucella* in tissues between groups was analyzed using a two-way ANOVA followed by Sidak’s or Tukey’s multiple comparisons test. Differences were considered significant if *P* value <0.05. Data are presented as standard error of mean (SEM). The HAI assay data is given as Geometric mean titer (GMT) with a confidence interval of 95%. Statistical analysis was performed using Graphpad Prism Software version 8.0 (Graphpad Software Inc., CA, USA).

## Results

### Vaccine Safety Assessment

#### General Clinical Status of Cattle After Vaccination

**Figure 2:**
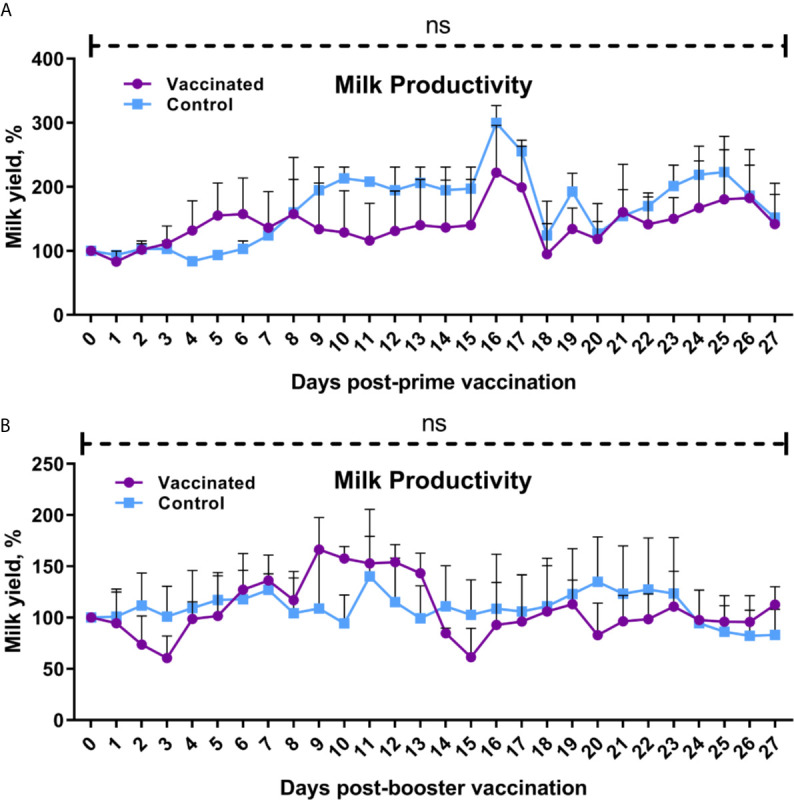
Cow milk yield after prime **(A)** and booster **(B)** vaccination. The cows in the vaccinated group were immunized twice *via* the subcutaneous route at an interval of 28 days with vaccines generated from the IVV subtypes H5N1 (prime vaccination) and H1N1 (booster vaccination). Cows in the control group were subcutaneously injected with 1.0 ml of 20% Montanide Gel01 adjuvant in PBS. Milk yield was expressed as a percentage of the initial volume of milk by 0 days before prime or booster vaccination. The data was presented as means with standard errors (SEM). Ns—*P = 0.1850 − > 0.9999 vs*. Zero-day milk yield prior to prime or booster vaccination; *P = 0.99 − > 0.9999* between vaccinated and control groups. Statistical analysis was performed using two-way ANOVA followed by Tukey’s or Sidak’s multiple comparisons test. *P* values < 0.05 were considered significant.

Clinical observation for 56 days after initial vaccination found no signs of disease including flu signs in animals, reduced appetite or abnormal behavior. Body temperature of calves, heifers and cows during the total 14-day observation period after prime and booster vaccination was within the physiological range (data not shown). No abortions were observed in pregnant heifers (n = 4) or cows (n = 6) have vaccination. No loss or significant (P = 0.1850 to >0.9999 *vs*. yields by 0 days after prime or booster vaccination) decrease in milk productivity of dairy cows was observed in both the experimental and control groups after prime and booster vaccination compared to baseline (**Figure 2**) and between groups (P = 0.99 to >0.9999).

#### Local Adverse Reaction in Cattle After Vaccination

**Figure 3:**
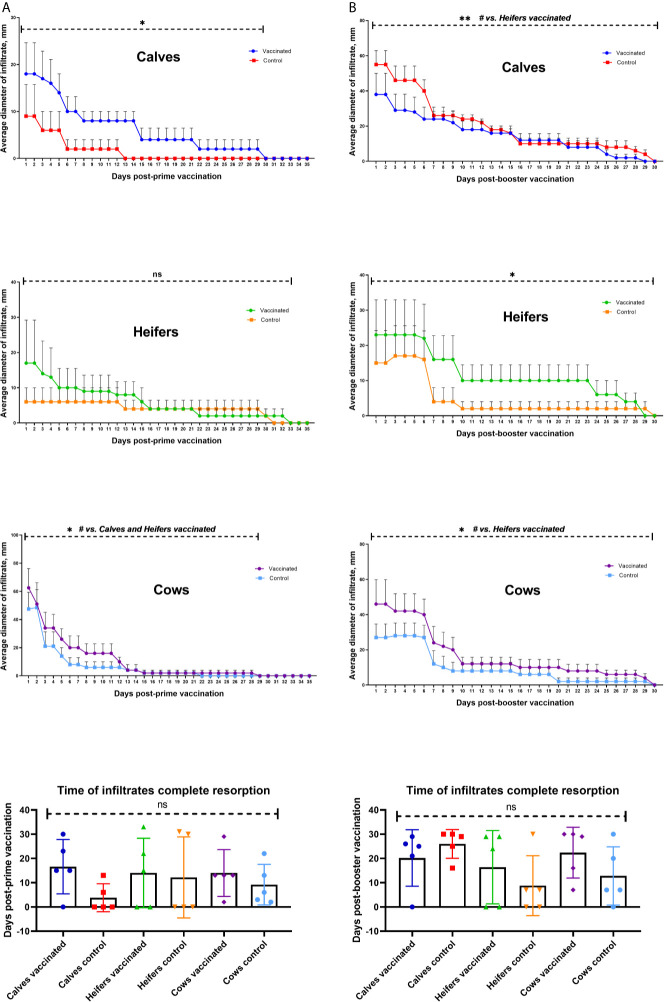
Dynamics of formation and resorption of infiltrates at the site of vaccination of different age groups of cattle within 35 days after prime **(A)** and 30 days after booster **(B)**. The calves, heifers, and adult cows in the vaccinated group were immunized twice *via* the subcutaneous route at an interval of 28 days with vaccines generated from the IVV subtypes H5N1 (prime vaccination) and H1N1 (booster vaccination). Animals in the control group were subcutaneously injected with 1.0 ml of 20% Montanide Gel01 adjuvant in PBS. Average infiltrate diameters were expressed in mm. The data was presented as means with standard errors (SEM). *P = 0.0025 − 0.0005 vs. appropriate control group; **P = 0.0033 vs. Calves vaccinated; ^#^ *P = 0.0025 − < 0.0001*; ns—*P = 0.25 − > 0.*9999. Statistical analysis was performed using two-way ANOVA followed by Tukey’s multiple comparisons test. *P* values < 0.05 were considered significant.

In 21/30 (70%) of cattle in both experimental and control groups, local inflammatory responses were detected at the injection site. In vaccinated animals, Groups I, II, and III had inflammatory responses detected in 80, 60, and 100%, respectively. In comparison, animals in control treatments receiving only adjuvant in PBS had detectable inflammatory responses at 40, 40, and 100%, respectively for in Groups I, II and III. The measured diameter of the inflammatory reaction was greatest in vaccinated adult cows (P <0.0001) (**Figure 3A**). Vaccinated cattle in calves and cows, but not heifers, had greater inflammatory responses than corresponding control groups (P <0.05). Average time for complete resorption in calves was 16.6 ± 5 days for vaccinated and 3.8 ± 2.5 days for controls, in heifers, 14.0 ± 3.6 days for vaccinates and 12.2 ± 7.4 days control group and cows, 14.0 ± 3.4 days experimental, 9.2 ± 3.7 days control group. There was no difference (P >0.05) between vaccinated and control treatments in the time for resolution of inflammatory responses (**Figure 3A**).

After booster vaccination, 24/30 (80%) of cattle, both experimental and control groups, infiltrates were formed at the site of injection one day after the vaccination. Formation of infiltration in cattle groups was as follows: calves, 80% experimental, 100% control group; heifers, 60% experimental, 60% control group; cows, 100% experimental, 80% control group. The largest (P = 0.0025 to <0.0001 *vs.* heifers) mean diameter of infiltrates was observed in adult experimental cows and calves of control groups (**Figure 3B**). Vaccinated heifers and cows had larger infiltration diameters than the corresponding control groups (P <0.0001), while calves had a reverse phenomenon (P = 0.0033 *vs.* experimental calves). The formed infiltrates were actively absorbed within 7 days after the booster vaccination. The average term of complete resorption of infiltrates by groups was as follows: calves 20.2 ± 5.21 days experimental, 26.0 ± 2.6 days control group; heifers, 16.4 ± 6.7 days experimental, 8.8 ± 5.5 days control group; cows, 22.4 ± 4.6 days experimental, 12.8 ± 5.3 days control group. No statistically significant difference was observed in the time of infiltrate resorption (**Figure 3B**) between experimental and control groups of animals, as well as between the age groups of cattle (P value from 0.34 to >0.9999).

#### Assessing the Persistence of the Vaccine Virus in Vaccinated Animals

PCR of nasal swabs obtained at 1 to 5 days and at 21 days after prime and booster vaccination, and all milk samples were negative for influenza type A virus (data not shown). Attempts to isolate virus in CE (two consecutive passages) were also no successful (data not shown).

#### Determination of Antibodies to Influenza Viral Vectors

**Figure 4:**
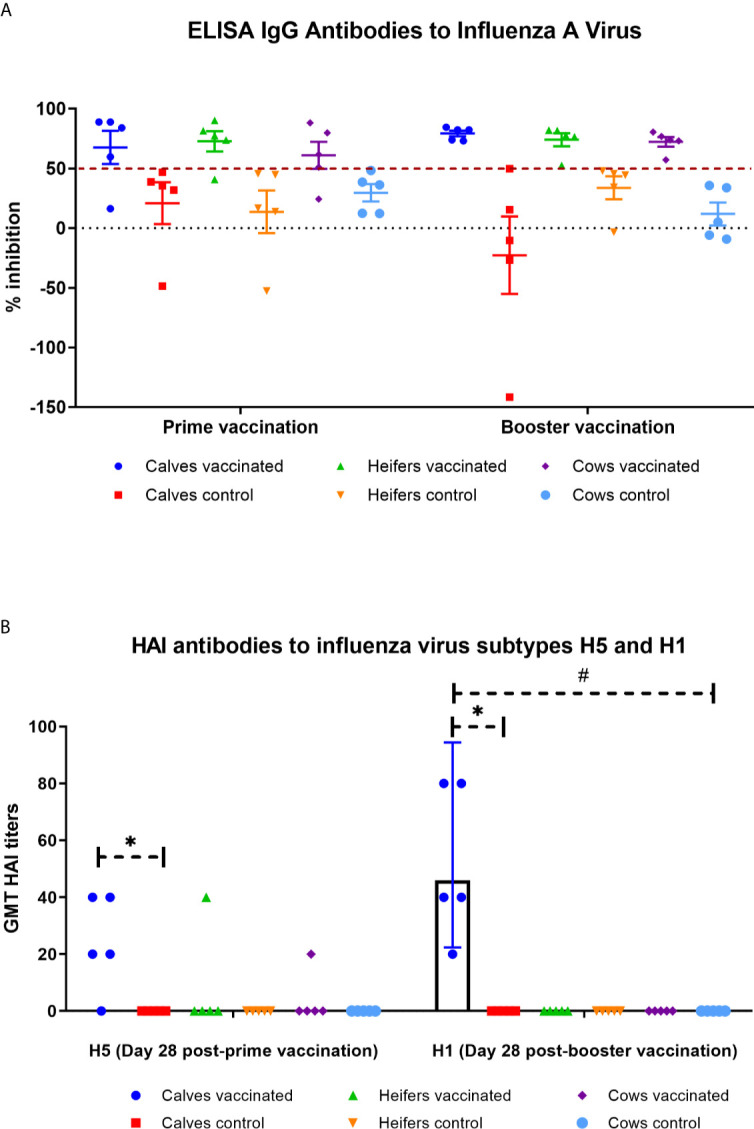
IgG antibodies to influenza virus type A by ELISA **(A)** and hemagglutinin inhibition (HAI) antibodies **(B)** to influenza virus subtypes H5 and H1 **(B)** in calves, heifers and cows for 28 days after prime and booster vaccination. The calves, heifers, and adult cows in the vaccinated group were immunized twice *via* the subcutaneous route at an interval of 28 days with vaccines generated from the IVV subtypes H5N1 (prime vaccination) and H1N1 (booster vaccination). Animals in the control group were subcutaneously injected with 1.0 ml of 20% Montanide Gel01 adjuvant in PBS. ELISA results were considered positive for optical density inhibition >50%. The HAI assay data is given as Geometric mean titer (GMT) with a confidence interval of 95%. *P = 0.0118 *−* P < 0.0001 *vs.* appropriate controls; ^#^P < 0.0001 *vs.* heifers and cows. Statistical analysis was performed using two-way ANOVA followed by Tukey’s multiple comparisons test. *P* values < 0.05 were considered significant.

In Groups I, II, and III, 4/5 (80%) demonstrated greater than 50% inhibition to influenza A virus on the ELISA assay (**Figure 4A**) at 28 days after prime vaccination. In a similar manner, in Groups I, II, and III at 28 days after booster vaccination the number of animals positively reacted to influenza A virus in the experimental groups reached 100% (**Figure 4A**). After booster vaccination, the average level of ELISA inhibition (60.9–72.7% *vs.* 72.3–79.2%) for influenza A slightly increased but did not have a statistically significant difference (P = 0.99 − >0.99 *vs.* prime vaccination). In the control group, all animals reacted negatively to influenza type A viruses.

Further examination of serum samples of the experimental groups showed that only calves had a significant (P = 0.0118 − P <0.0001) accumulation of HAI antibodies to influenza virus subtype H5 or H1 in comparison with the control groups (**Figure 4B**). The level of accumulation of antibodies to influenza virus subtype H1 was significantly higher (P = 0.001) than that of H5. Moreover, GMT titer of HAI antibodies to influenza subtype H1 virus in vaccinated calves was significantly higher (P <0.0001) than those of heifers and cows (they had no HAI antibodies at all). No HAI antibodies to influenza virus subtype H5 and H1 were found in the serum of control animals during the observation period.

In all groups of animals at 0 days of prime vaccination, no IVV antibodies in ELISA or HI assay were detected (data not shown).

### Differentiation of Infected From Vaccinated Animals

Serum from vaccinated cattle obtained at 28 days after prime and booster vaccination was negative on RBA, SAT and ELISA kits (data not shown).

### Determination of Vaccine Immunogenicity

**Figure 5:**
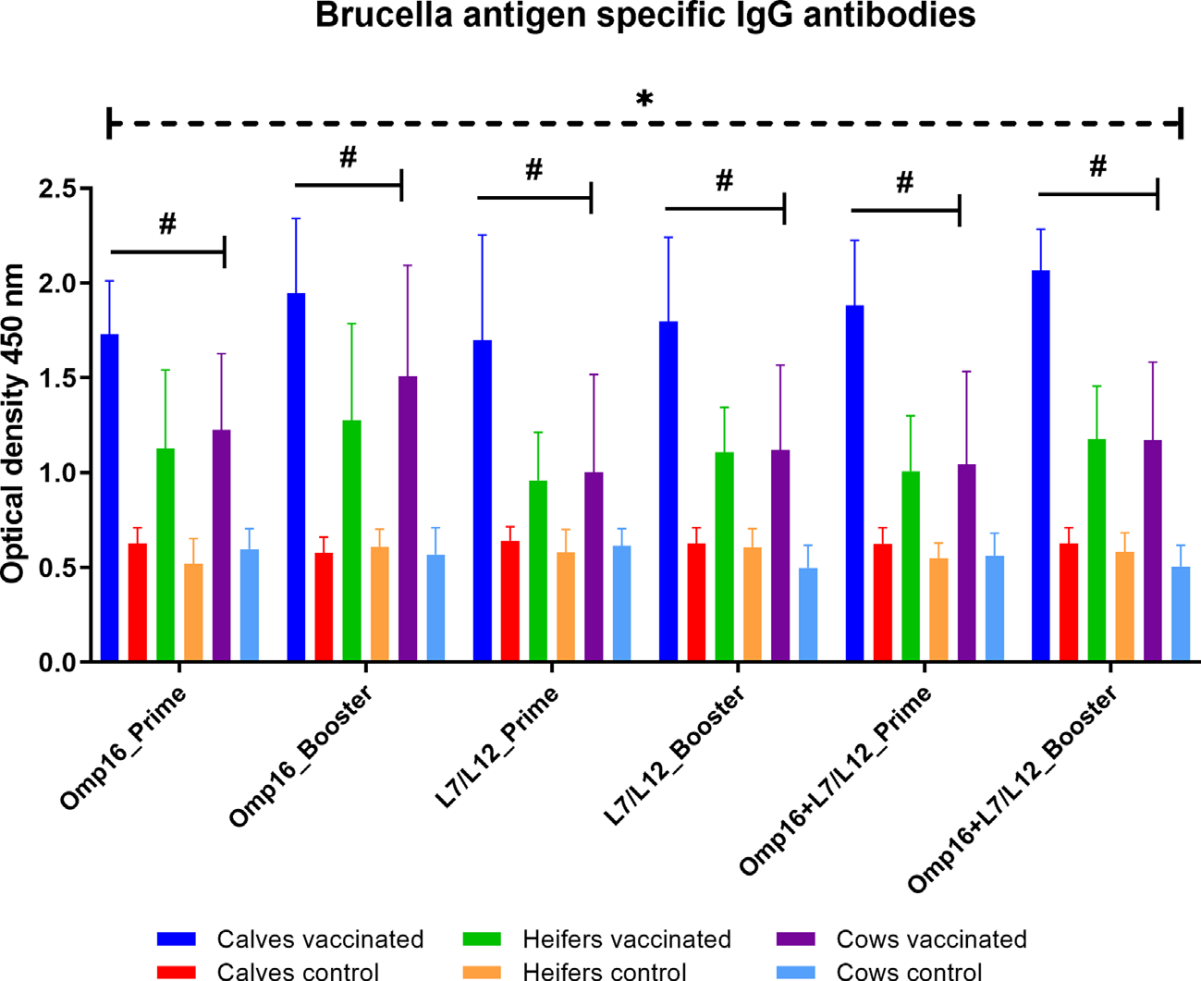
IgG antibodies to individual *Brucella* Omp16 or L7/L12 proteins and to their mixtures by ELISA in calves, heifers and cows for 28 days after prime and booster vaccination. The calves, heifers, and adult cows in the vaccinated group were immunized twice *via* the subcutaneous route at an interval of 28 days with vaccines generated from the IVV subtypes H5N1 (prime vaccination) and H1N1 (booster vaccination). Animals in the control group were subcutaneously injected with 1.0 ml of 20% Montanide Gel01 adjuvant in PBS. The data was presented as means with standard errors (SEM). *P = 0.04 – P < 0.0001 *vs.* appropriate controls; ^#^P = 0.01 – P < 0.0001 *vs.* heifers and cows. Statistical analysis was performed using two-way ANOVA followed by Tukey’s multiple comparisons test. P values < 0.05 were considered significant.

When compared to controls, vaccinated animals in all groups had antibodies to Omp16 and L7/L12 proteins for 28 days after prime and booster vaccination (**Figure 5**). Among the different age groups of cattle, the highest accumulation of antibodies to both individual and mixed Omp16 and L7/L12 proteins was noted in calves (P = 0.01 – P <0.0001 *vs.* heifers and cows) after prime and booster immunization. It should be noted that booster vaccination of calves, heifers and cows contributed only to a slight (P = 0.58–0.97 *vs.* prime vaccination) increase in the IgG antibody levels to *Brucella* proteins.

### Challenge of Cattle to Assess Vaccine Protectiveness

**Figure 6:**
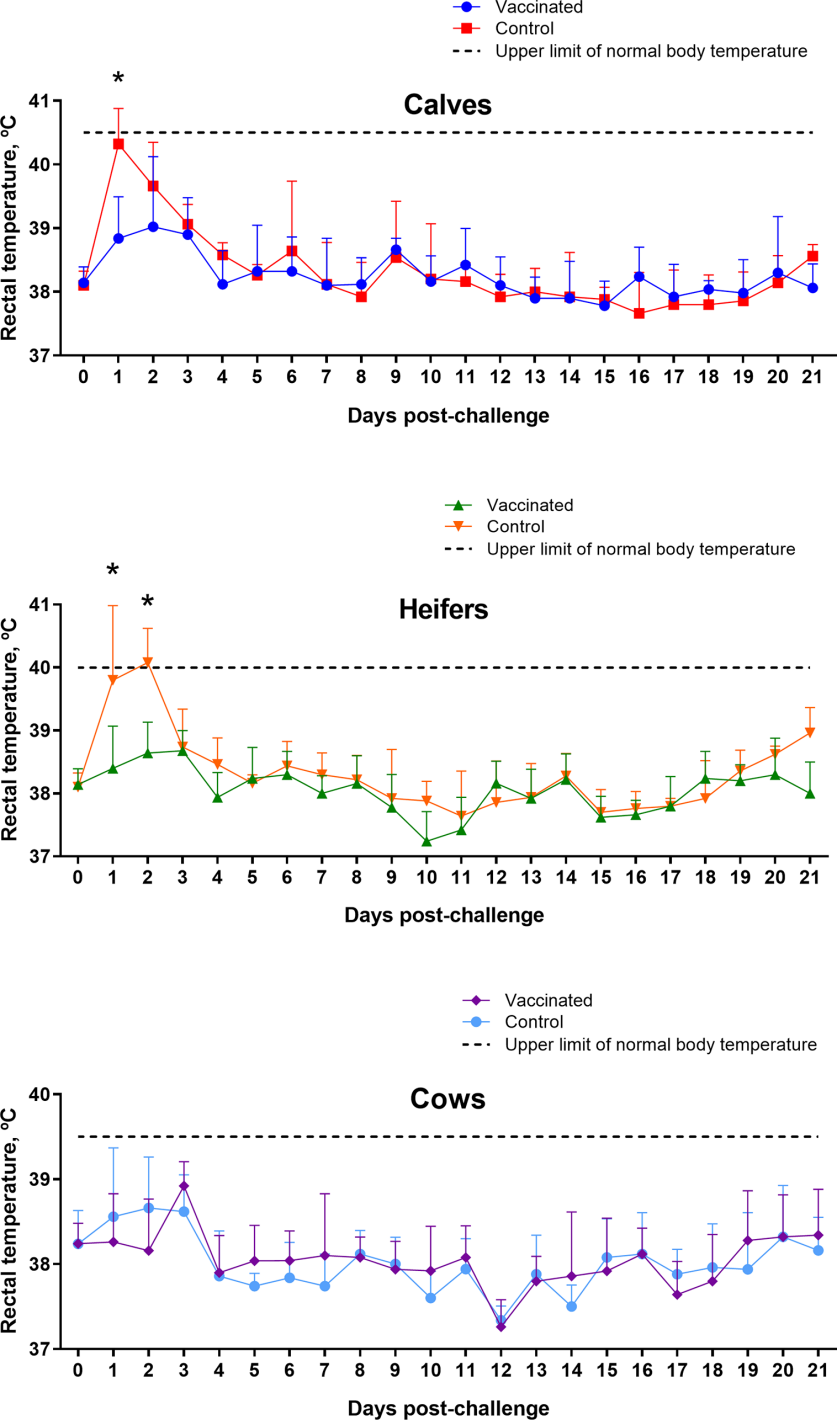
Rectal temperature of calves, heifers and cows of experimental and control groups after challenge with virulent strain *B*. *abortus* 544. The calves, heifers, and adult cows in the vaccinated group were immunized twice *via* the subcutaneous route at an interval of 28 days with vaccines generated from the IVV subtypes H5N1 (prime vaccination) and H1N1 (booster vaccination). Animals in the control group were subcutaneously injected with 1.0 ml of 20% Montanide Gel01 adjuvant in PBS. All the animals of the experimental and control groups were subjected to virulent strain *B. abortus* 544 challenge infection at 28 days after booster vaccination. Body temperature was measured at 0–21 days after challenge. Normal body temperature: calves—38.5–40.5°C; heifers—38.5–40.0°C; cows—37.5–39.5°C. The data were presented as means with standard errors (SEM). *P = 0.009 – P < 0.0001 *vs*. appropriate vaccinated groups. Statistical analysis was performed using two-way ANOVA followed by Sidak’s multiple comparisons test. P values < 0.05 were considered significant.

The results of a 30-day clinical observation of challenged animals in Groups I–III did not reveal any clinical signs of brucellosis. In particular, due to the short period of observation, there were no abortions in both control and vaccinated pregnant heifers (approximately 3.5–5 months of pregnancy). The body temperature of calves, heifers and cows in the experimental groups was within the limits of physiological range during the 21-day observation, however, in calves and heifers in the control groups at 1 and 1–2 days after the control infection, respectively, a significant (P = 0.009 – P <0.0001 *vs.* appropriate vaccinated groups) increase in body temperature was observed (**Figure 6**).

The results of clinical observation of infected pregnant cows (Group III), which lasted 6.5 months, showed that vaccination provides 100% (3/3) protection against abortion (**Table 2**). All vaccinated cows were successfully calved after 5.5–6.5 months (depending on the duration of pregnancy) of challenge *B. abortus* 544 infection. In contrast, in the control group, all (3/3) cows were aborted 3.5–6 months after challenge.

**Table 2:** Abortion, calving and infection rates in different age groups of cattle after challenge with virulent strain *B. abortus* 544 infection.

Group	Aborted/Calved, number*	Number of *Brucella* isolated animals, Positive/Negative	Vaccine effectiveness	Number of *Brucella* isolated fetuses and calves, Positive/Negative	Total
**Calves vaccinated**	–	1/4	75 %	–	5
**Calves control**	–	4/1**#**		–	5
**Heifers vaccinated **	–	2/3	60 %	0/2	5
**Heifers control**	–	5/0		0/2	5
**Cows vaccinated**	0/3	2/3	60 %	0/3	5
**Cows control**	3/0	5/0		3/0	5

*Aborted cows also included those who gave birth to non-viable calves.

^#^In one bull in the control group Brucella was not isolated after challenge.

All isolates were identified as B. abortus.

**Figure 7:**
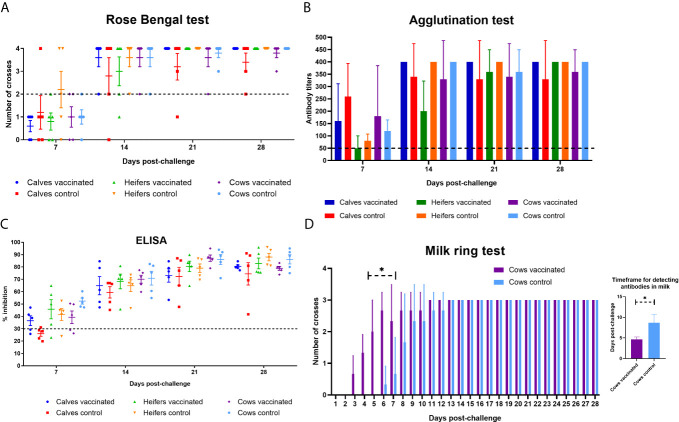
Evaluation of calves, heifers and cows in experimental and control groups for brucellosis by Rose Bengal test **(A)**, serum agglutination test **(B)**, ELISA **(C)** and milk ring test **(D)** within 28 days after challenge with *B. abortus* 544. The calves, heifers, and adult cows in the vaccinated group were immunized twice *via* the subcutaneous route at an interval of 28 days with vaccines generated from the IVV subtypes H5N1 (prime vaccination) and H1N1 (booster vaccination). Animals in the control group were subcutaneously injected with 1.0 ml of 20% Montanide Gel01 adjuvant in PBS. All the animals of the experimental and control groups were subjected to virulent strain *B. abortus* 544 challenge infection at 28 days after booster vaccination. The results of the assay reactions were considered positive: RBT in the presence of pronounced agglutination (on two to four crosses); SAT in the presence of agglutination in the serum dilution 1:50 and more; ELISA in the inhibition of OD > 30%; milk ring test in the presence of agglutination (one to three crosses). The data was presented as means with standard errors (SEM). *P = 0.03 – P < 0.0001 *vs.* appropriate controls. Statistical analysis was performed using two-way ANOVA followed by Sidak’s multiple comparisons test or t-test. P values < 0.05 were considered significant.

Almost all animals in the experimental vaccinated and control groups reacted positive to brucellosis in RBA, SAT and ELISA from 7 days after challenge infection (**Figures 7A–C** **)**, and higher antibody reactivity was observed at 21–28 days after challenge. However, it is noteworthy to mention that in the milk of vaccinated cows (n = 3) antibodies in the ring test were formed significantly earlier (4.6 ± 0.5 *vs.* 8.6 ± 2.0 days; P = 0.03) than in the control group (n = 3) after challenge infection (**Figure 7D**). At the time of calving or abortion, cows in the experimental and control groups were still seropositive for brucellosis in RBA, SAT and ELISA (data not shown).

**Figure 8:**
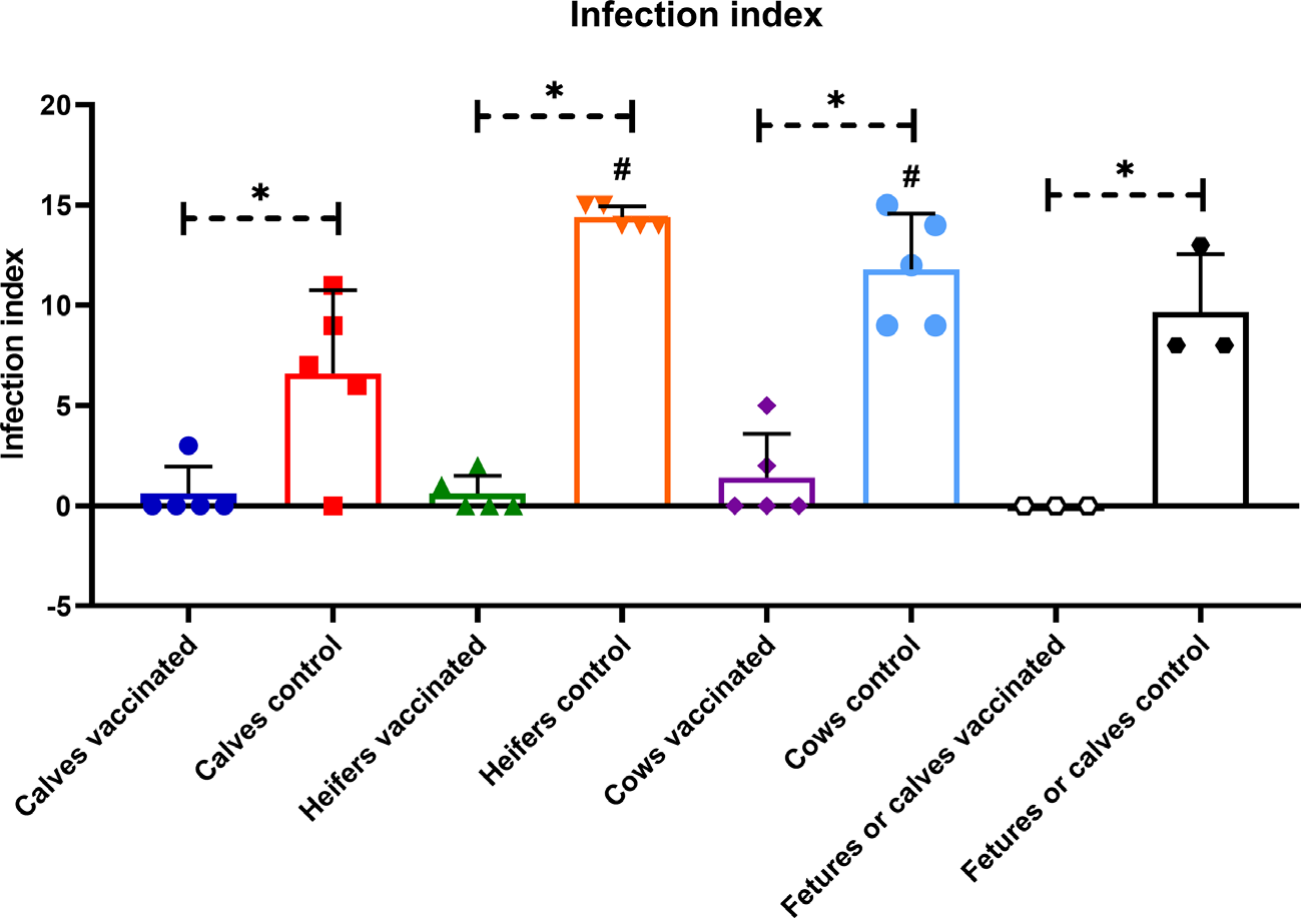
Infection index of calves, heifers, cows, and their calves or aborted fetuses of the experimental and control groups after challenge with *B. abortus* 544 infection. The calves, heifers and adult cows in the vaccinated group were immunized twice *via* the subcutaneous route at an interval of 28 days with vaccines generated from the IVV subtypes H5N1 (prime vaccination) and H1N1 (booster vaccination). Animals in the control group were subcutaneously injected with 1.0 ml of 20% Montanide Gel01 adjuvant in PBS. All the animals of the experimental and control groups were subjected to virulent strain *B. abortus* 544 challenge infection at 28 days after booster vaccination. Infection index—number of organs and lymph nodes of animals in which *Brucella* were isolated. The data was presented as means with standard errors (SEM). *P = 0.04 – P < 0.0001 *vs.* appropriate controls; ^#^P = 0.009 – P < 0.0001 *vs*. calves control. Statistical analysis was performed using two-way ANOVA followed by Tukey’s multiple comparisons test. P values < 0.05 were considered significant.

**Figure 9:**
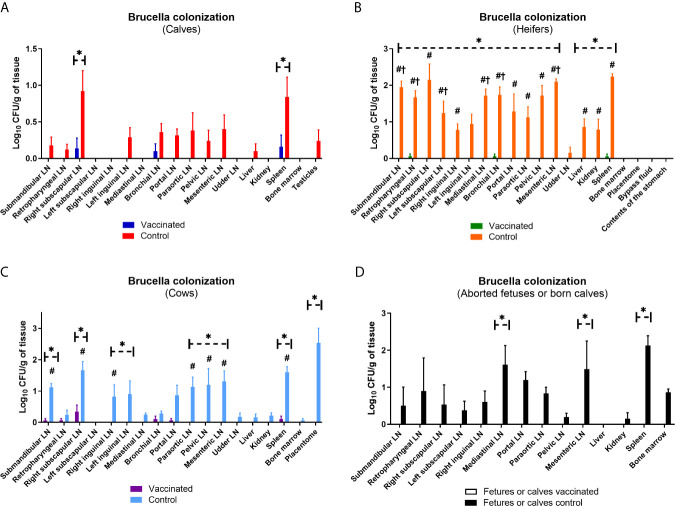
Level of Brucella colonization from the lymph nodes and organs of calves **(A)**, heifers (including their fetuses) **(B)**, cows **(C)**, and their calves or aborted fetuses **(D)** of the experimental and control groups after challenge with B. abortus 544. The calves, heifers, and adult cows in the vaccinated group were immunized twice via the subcutaneous route at an interval of 28 days with vaccines generated from the IVV subtypes H5N1 (prime vaccination) and H1N1 (booster vaccination). Animals in the control group were subcutaneously injected with 1.0 ml of 20% Montanide Gel01 adjuvant in PBS. All the animals of the experimental and control groups were subjected to virulent strain B. abortus 544 challenge for 28 days after booster vaccination. The degree of Brucella colonization in lymph nodes and organs was expressed in Log10 CFU/g tissue. The data was presented as means with standard errors (SEM). *P = 0.04 P < 0.0001 vs. appropriate controls; ^#^P = 0.04 P < 0.0001 *vs.* calves control; ^†^P = 0.03 − 0.0003 *vs.* cows control. Statistical analysis was performed using two-way ANOVA followed by Sidak's multiple comparisons test. P values < 0.05 were considered significant.

Prime booster immunization with Flu-BA provided 75, 60 and 60% effectiveness in calves, heifers and cows, respectively, against experimental challenge infection with *B. abortus* 544 (**Table 2**). Interestingly, the sensitivity level of calves to brucellosis (infection index P = 0.009 – P <0.0001, **Figure 8**; *Brucella* colonization from tissues P = 0.04 – P <0.0001; **Figure 9**), including bulls (one of them had no *Brucella* at all), was generally significantly lower than that of heifers and cows. At the same time, the maximum level of infection index and the *Brucella* colonization from tissues after challenge (P = 0.03 − 0.0003 *vs.* cows control; **Figure 9**) were observed in the heifers of the control group, including pregnant heifers, in comparison with those of calves and cows.

The level of complete protection against *B. abortus* 544 fetal infection (n = 2) of vaccinated pregnant heifers (4.5–7 months of pregnancy) and calves (n = 3) born from vaccinated cows was 100% (**Table 2**). However, in the control group, *Brucella* were isolated only in aborted fetuses or calves of cows (3/3 or 100%), but were not isolated from fetuses (n = 2) of pregnant heifers.

Severity of *B. abortus* 544 infection in groups of vaccinated calves, heifers and cows, as well as calves that were born to cows after challenge infection as estimated by an infection index (0 to 1.4; **Figure 8**). *Brucella* colonization in tissue samples of vaccinates was lower than (up to 0.34 Log10 CFU/g tissue; **Figure 9**) was significantly lower (P = 0.04 – P <0.0001) than colonization in tissues from the controls (*Brucella* colonization up to 2.53 Log10 CFU/g tissue). The index of infection and the degree of *Brucella* colonization from tissues after challenge infection between the experimental groups of different age did not have a significant difference (P = 0.99 − P >0.99). It should be noted that in vaccinated bulls (n = 2), in contrast to control animals (2/3 or 66%) no *Brucella* was isolated in the testicles.

## Discussion

This work was a continuation of our series of studies started in 2012 aimed at developing and characterizing the novel *B. abortus* vaccine by using the IVV platform. In this study, we examined the safety and efficacy of the Flu-BA vaccine compared simultaneously in different age groups of cattle, including pregnant animals. These studies were carried out as part of the Flu-BA vaccine registration trial in Kazakhstan, and therefore they were carried out according to the requirements and supervision of the regulatory body with the involvement of leading experts in this field. At the request of the Regulatory Body, this study not only required confirmation of the previously stated properties of the vaccine on cattle, but also disclosure of the properties of the vaccine described in this paper. In particular, it was necessary to determine the effectiveness of the vaccine in young cattle (from 4 months of age). This was because all known commercial brucellosis vaccines (*B. abortus* S19 and RB51) have been in use in young animals as per manufacturer’s instructions. It was also interesting to study the effectiveness of the vaccine in bulls. This requirement was justified by the fact that bulls with brucellosis are able to spread the infection through semen since the organisms predominantly infect testicle (Gemechu, 2017), although in general they do not play a significant role in epidemiology of the disease. Another important interest was study of the degree of development of influenza infection in cattle vaccinated with IVV, which in this case was used as a carrier to deliver foreign genes into the host cells. This interest was because influenza A viruses in sporadic cases infect cattle and cause signs of influenza infection (Campbell et al., 1977; Brown et al., 1998; Gunning et al., 1999; Graham et al., 2002).

In the first series of studies, we comparatively investigated the safety of Flu-BA vaccine in different age groups of cattle (including pregnant ones), assessing both general and local adverse events. Overall, studies have shown that the tested vaccine formulation (IVV suspension subtype H5N1 or H1N1-stabilizing media-adjuvant), regardless of age and pregnancy status of the cattle, is completely safe and not abortogenic (in both pregnant heifers and adult cows). The only aspect is that the vaccine was not devoid of local adverse effects, which was characterized by the formation of infiltrates in the area of injection. This was due to use of the polymeric adjuvant Montanide Gel01 as the control group of animals received only that adjuvant had an identical reaction. It is noteworthy to mention here that in this study we showed for the first time the dynamics of formation and complete resorption of subcutaneous infiltrates in cattle after prime and booster vaccination. Based on the results of these studies, we were unable to identify any logical dependence of this indicator on the age status of cattle. The only thing that was obvious is that after prime and booster vaccination, the largest infiltrates was observed in the experimental group of cows. At the same time, there was no difference in the time of complete resorption of infiltrates between the groups. Complete resorption of infiltrates in all age groups of cattle took place within 35 days after each vaccination, which is fully consistent with the Flu-BA vaccine instructions. Despite the fact that the described local adverse effect of the Montanide Gel01 adjuvant, its use in the vaccine formulation (as a diluent) to increase the effectiveness of the preparation is fully justified. In general, the results are fully consistent with our previous studies (Tabynov et al., 2014a; Tabynov et al., 2016a).

Separate attention was given to the safety aspects of the vaccine as IVV used may cause influenza infection in cattle upon Flu-BA vaccination. Cattle are generally not susceptible to influenza type A virus, but there are reports of sporadic outbreaks of human influenza virus (strains A/Eng/333/80 H1N1 and A/Eng/427/88 H3N2) infection in dairy cows (Gunning et al., 1999; Graham et al., 2002). Clinical manifestation of the infection was expressed by virus in the nose, but most often by a dramatic fall in milk yield, which was recovered only 1–2 weeks after the infection. Retrospective analysis of cow serum samples in infected herds showed HAI antibodies to influenza virus subtypes H1 and H3 in about 50% of animals with the titer 1:10–1:640 (Graham et al., 2002). In our studies, no age dependent including in lactating pregnant animals any influenza signs after prime and booster vaccination with Flu-BA. No loss or significant decrease in milk production was observed in dairy cattle after vaccination. Moreover, vaccine viruses were not detected in nasal swabs and milk of vaccinated animals. We attribute this to the subcutaneous method of vaccination and secondly to the limited replication ability of IVV due to truncated interferon antagonist NS1 protein (replacing polybasic cleavage site with a trypsin-dependent sequence in IVV subtype H5). The only response of all age groups of cattle to the IVV vaccination was the formation of IgG antibodies to the influenza A virus, and in calves the formation of HAI antibodies to the influenza virus subtypes H5 and H1 was predominant. Based on results of these studies we conclude that Flu-BA vaccine does not cause influenza signs of infection and it is safe for use in cattle.

We then experimentally confirmed Flu-BA’s compliance with the DIVA criteria by examining serum of cattle vaccinated by using routine serologic brucellosis diagnostic tests—RBT, SAT and ELISA. As expected, animals vaccinated with Flu-BA vaccine reacted negatively in these tests. This is due to the fact that all brucellosis diagnostic tests are mainly aimed at detecting *Brucella* anti-OPS antibodies, while IVV, expressing *Brucella* Omp16 and L7/L12 proteins failed to react. The results obtained are fully consistent with our previous studies (Tabynov et al., 2014a; Tabynov et al., 2016a). Thus, Flu-BA vaccine meets the important criteria of DIVA, which in turn allows for a successful vaccination campaign.

Another research blog was devoted to study of the antibody response to Flu-BA vaccine in different age groups of cattle. It should be noted that, unlike the previous studies (IgG, IgG1, IgG2a antibodies; lymphocyte stimulation index, gamma interferon production) (Tabynov et al., 2014d; Tabynov et al., 2016c), here the study of vaccine immunogenicity was limited to the detection of *Brucella* antigen-specific IgG antibodies by ELISA. This was because Flu-BA vaccine guidelines stated that the only way to evaluate the vaccine effectiveness on both model animals (mice and guinea pigs) and cattle is through the challenge method. Therefore, detailed immunogenicity testing in animals was not required by the regulatory body. Our data showed that in all age groups of cattle for 28 days after prime and booster vaccination IgG antibodies to both individual proteins Omp16 and L7/L12 and their mixtures were significantly increased. This fact in itself indirectly indicates the expression of these *Brucella* proteins in the body of vaccinated animals, as well as the absence of interference between two IVVs expressing different *Brucella* proteins. It is noteworthy to mention that among different age groups of cattle the highest induction of antibodies to *Brucella* proteins after both prime and booster vaccination was observed in calves. Interestingly, these data are consistent with results of the antibody response to IVV, where the better induction of HAI antibodies were demonstrated in calves. It is important to note that after booster vaccination all experimental cattle had a slight increase in the level of *Brucella* antigen-specific IgG antibodies compared to after prime vaccination. This fact suggests that a cross-immunization scheme is justified to overcome the immune background to the viral vector, IVV with different subtypes of hemagglutinin were used (H5 for prime, H1 for booster vaccination).

The final and defining phase of work was to test Flu-BA vaccine protection in different age groups of cattle. The protection level of vaccinated animals against *B. abortus* 544 infection was assessed based on clinical observation (temperature response, monitoring of abortions) and bacteriological studies. The latter method allows not only the presence/absence of the pathogen in infected animal but also shows the degree of incidence (infection index) and intensity (the level of colonization of brucellosis from tissues) of brucellosis infection. The results of the studies showed that prime-booster subcutaneous immunization of cattle with Flu-BA vaccine provides complete protection against clinical manifestation of brucellosis infection; and the vaccinated pregnant heifers did not abort and the cows gave birth to healthy calves during the period of observation. All animals, both in the experimental and control groups, after challenge infection responded positively to brucellosis in RBA, SAT, ELISA, as well as in the milk ring test (dairy cows). It is interesting to note that in the milk of vaccinated cows antibodies in the ring test were formed significantly earlier (4.6 ± 0.5 *vs.* 8.6 ± 2.0 days after challenge) than in the control group. In our opinion, this is one of the evidence of a rapid immune response to the infectious agent in animals because of vaccination.

Bacteriological analysis of Flu-BA vaccine protection has shown that it provides 75% effectiveness against *B. abortus* 544 infection in immunized calves, including bulls, as well as 60% effectiveness in heifers and cows, including pregnant animals. Here, we showed that Flu-BA vaccine is more effective in calves than adult animals based on the immunogenicity data. Likewise, susceptibility of calves to brucellosis infection, including bulls, was significantly lower than that of heifers and cows. This is despite the fact that in all age groups of cattle the same dose of virulent strain *B. abortus* 544 was used. In general, the low susceptibility of young cattle to brucellosis can also be explained by the presence of bulls in this group of cattle, which are less susceptible than female cattle and in them brucellosis is predominantly manifested in the form of orchitis (Gemechu, 2017). It is important to note that the vaccinated bulls, unlike the control group, do not have *Brucella* in their testicles after challenge. These preliminary results indicate that the immunization of bulls with Flu-BA vaccine not only protects against *B. abortus* 544 infection, but also has the potential to prevent the spread of the pathogen through semen. With regard to heifers and cows, including pregnant ones, it can be noted that brucellosis infection was relatively rapid in these groups, with a high index of infection and *Brucella* colonization from tissues, as well as 100% abortions in pregnant cows. Therefore, the vaccination efficacy in heifers and cows was slightly lower than in calves. The level of effectiveness achieved in heifers and cows (60%) is comparable to previous studies (Tabynov et al., 2014e; Tabynov et al., 2016c), where the efficacy under similar conditions was in the range of 70–80%, and to the commercial RB-51 vaccine (50%) (Tabynov et al., 2016c). In general, there were no significant differences between vaccinated calves, heifers and cows in terms of the index of infection and degree of *Brucella* colonization in tissues. Thus, it can be concluded that Flu-BA vaccine is safe and effective in all age groups of cattle, and fully meets the requirements of regulatory documentation.

Based on results of this study, Flu-BA vaccine has been successfully tested on target animals, confirmed as previously stated (according to the instructions for use), and demonstrated new (at the request of the regulatory body) properties of the vaccine in cattle. The results of this study conducted in conjunction with other studies for compliance with the preparations specifications (physico-chemical and biological properties), made it possible to register it in the State Register of Veterinary Preparations and Feed Additives of the Ministry of Agriculture of the Republic of Kazakhstan (registration certificate No. RK-VP-1-3775-19 dated January 14, 2019). In spite of relatively short period of 6 years of complex research, we managed to develop not only a novel vaccine candidate but also thoroughly studied its properties in the target animals; most importantly, introduced for field use in cattle of all age and pregnancy status. The high importance of this work is the fact that Flu-BA is the first vaccine introduced in the last two decades after commercial *B. abortus* RB-51 vaccine. The main advantages of this vaccine in comparison with commercial vaccine was that our study eventually led to its registration in Kazakhstan (Tabynov, 2016). It is obvious that Flu-BA vaccine by safety profile (absence of any temperature reaction, abortions, persistence of vaccine viruses in nose and shedding through milk) significantly exceeds all known commercial brucellosis vaccines. In addition, it does not present any serious risk to human health (Ferko et al., 2004) unlike other commercial *Brucella* vaccines (Spink et al., 1962; Beckett and MacDiarmid, 1985; Schurig et al., 1991). This is evidenced by the fact that research is currently under way using IVV platform to develop a brucellosis vaccine for humans (Bugybayeva et al., 2020; Bugybayeva et al., 2021). Another important advantage of Flu-BA vaccine is that it is in line with the DIVA strategy, which allowed *B. abortus* RB-51 to virtually replace the earlier *B. abortus* S19 vaccine from the market. Commercial vaccines, *B. abortus* S19 and RB51 (according to their application instructions), do not differ significantly from Flu-BA in their basic immunobiological properties (timing and duration of the protective immune response, effectiveness of vaccine and expiration date). Although, in contrast to *B. abortus* RB51 vaccine with lack of information, our vaccine product is capable of providing cross-protection in vaccinated cattle against *B. melitensis* infection (Tabynov et al., 2015). This feature of the vaccine is particularly critical in developing and under developed *Brucella* endemic countries wherein small ruminants and cattle are reared together. Flu-BA is significantly more expensive than *B. abortus* S19 (almost four times), although it is almost three times cheaper than the most common and most popular *B. abortus* RB-51 vaccine. We hope that the combination of these benefits of Flu-BA vaccine, which meets most of the criteria for “ideal brucellosis vaccine” as defined by Ko and Splitter (2003) will eventually allow it to gain a foothold in the veterinary market. Currently, Flu-BA vaccine is undergoing post-registration testing (field trials) at the initiative of the manufacturer; in particular, it is used to recover several livestock farms in Kazakhstan with different levels of prevalence of brucellosis infection. If successful at this stage, large-scale production and use of this vaccine is planned in Kazakhstan, and possibly in other countries.

## Conclusion

Our trials of Flu-BA vaccine in different age groups of cattle, including pregnant cattle upon prime and booster subcutaneous immunization showed complete safety profile and not abortogenic. But it exhibits moderate local adverse events in the form of development of infiltrates at the injection site, which completely gets resorbed within 35 days after vaccination. Furthermore, the vaccine did not cause any signs of influenza infection, reduction or loss of milk production in dairy cattle and absence of persistence of IVV in vaccinated animals in nose and milk. It does not elicit antibodies that respond positively to routine serologic brucellosis diagnostic tests, and therefore meets the DIVA criterion. The vaccine induces specific antibodies to *Brucella* Omp16 and L7/L12 proteins, with the highest response in calves. Flu-BA vaccine provides full protection against clinical manifestations of brucellosis, including abortion, and 75, 60 and 60% effectiveness against *B. abortus* 544 infection in immunized calves (including bulls), heifers and cows (including pregnant ones). The vaccine provides 100% protection against *B. abortus* 544 infection of calves from immunized pregnant cows. To conclude the Flu-BA vaccine has now passed the registration commission tests of safety and protection on different age groups of cattle, including pregnant animals, and was therefore recommended for practical use in all age group of cattle.

## Data Availability Statement

The original contributions presented in the study are included in the article/supplementary material. Further inquiries can be directed to the corresponding author.

## Ethics Statement

The animal study was reviewed and approved by the Committee on the Ethics of Animal Experiments of the Research Institute for Biological Safety Problems of the Science Committee of the Ministry of Education and Science of the Republic of Kazakhstan.

## Author Contributions

Conceptualization: KaisT. Methodology: KaisT, SR, NZ and BA. Validation: BK, AB, BA, KA and AS. Formal Analysis: KaisT, GR and KairT. Investigation: SR, NZ, ZK, BY, YK, DI, NA, AM, DB and MS. Resources: KaisT and TY. Data Curation: SR. Writing—Original Draft Preparation: KaisT. Writing—Review and Editing: KaisT, GR, SO and AO. Visualization: KaisT and KairT. Supervision: BK, AB, BA, KA and AS. Project Administration: KaisT, BK and AS. Funding Acquisition: KaisT. All authors contributed to the article and approved the submitted version.

## Funding

This research has been funded by the Ministry of Agriculture of the Republic of Kazakhstan (BR10764975) and the Institute for Biosafety Research's own budget, as well as with the partial financial support of Seppic (France). The funders were not involved in the study design, collection, analysis, interpretation of data, the writing of this article or the decision to submit it for publication.

## Conflict of Interest

The authors declare that the research was conducted in the absence of any commercial or financial relationships that could be construed as a potential conflict of interest.
